# Non-Layered Gold-Silicon and All-Silicon Frequency-Selective Metasurfaces for Potential Mid-Infrared Sensing Applications

**DOI:** 10.3390/s21165600

**Published:** 2021-08-19

**Authors:** Octavian Dănilă, Doina Mănăilă-Maximean, Ana Bărar, Valery A. Loiko

**Affiliations:** 1 Physics Department, ‘Politehnica’ University of Bucharest, 313 Splaiul Independentei, 060042 Bucharest, Romania; doina.manaila@upb.ro; 2 Electronic Technology and Reliability Department, ‘Politehnica’ University of Bucharest, 313 Splaiul Independentei, 060042 Bucharest, Romania; ana.barar@upb.ro; 3 B. I. Stepanov Institute of Physics, National Academy of Sciences of Belarus, 68-2 Nezavisimosti Ave., 220072 Minsk, Belarus; loiko@ifanbel.bas-net.by

**Keywords:** hybrid metasurfaces, frequency selective surfaces, infrared sensors, electromagnetic wave control

## Abstract

We report simulations on the spectral behavior of non-layered gold-silicon and all-silicon frequency-selective metasurfaces in an asymmetric element configuration in the mid-infrared spectral window of 5–5.8 μm. The non-layered layout is experimentally feasible due to recent technological advances such as nano-imprint and nano-stencil lithography, and the spectral window was chosen due to the multitude of applications in sensing and imaging. The architecture exhibits significant resonance in the window of interest as well as extended tunability by means of variation of cell element sizes and relative coordinates. The results indicate that the proposed metasurface architecture is a viable candidate for mid-infrared absorbers, sensors and imaging systems.

## 1. Introduction

Metasurfaces [1,2,3], are relatively new and promising alternatives to conventional metamaterials [4,5,6] in shaping, manipulation and conversion of electromagnetic radiation across a wide range of spectra. Just like metamaterials, metasurfaces are two-dimensional periodic elements of imagined geometries and materials that interact with the electromagnetic field in such a way that the response is no longer given by the atoms of the structure but rather by the effective meta-atoms that are created by combining a desired geometry with specific materials. As a rule of thumb, meta-atoms having an average geometric dimension *a* will resonate with an optical field having a wavelength $\lambda \in \left(5a,10a\right)$ with specific responses being tailored by the actual geometry and the electromagnetic properties of the composing materials [7]. While initially designed for GHz to THz frequencies due to limitations in the cell manufacturing processes, the advancement of technological methods for deposition, lithography and printing have pushed the design of metasurfaces into the mid-infrared and visible spectrum [8,9]. The ability to scale down the unit cell has led to an extended set of applications in optics, in the form of novel reflection and refraction materials [10], frequency-selective surfaces across a wide variety of spectra [11], optical cloaking [12,13], biological sensors [14], holography [15,16], polarization handling [17], broadband and achromatic devices [18], diffraction [19] and wavefront manipulation [20], as well as non-trivial effects such as photonic spin Hall effects [21], photovoltaics [22], orbital angular momentum generation [23], harmonics generation [24], optical bifunctionality [25] and quantum informational effects [26,27]. In the mid-infrared domain, also known as the ‘molecular fingerprint’ window, their chirality has enabled metasurfaces to be used in high-sensitivity molecular spectroscopy [28]. In addition, their frequency-selective behavior has been exploited in the realization of mid-infrared cameras and detectors [29], with promising results exhibited by low-loss phase change materials such as aluminum [30] and, more recently, chalcogenides such as GeSbTe alloys [31,32,33,34,35]. The mid-infrared domain is also a window of interest for single-gas and gas mixtures concentration sensing [36,37], with integrated solutions realized by means of waveguide-based point spectrometers [38], functionalized microring nanoantennas [39] and more recently, nanoantenna-based chips [40]. In such applications, metasurfaces have been used as tunable bandpass filters that are able to select the wavelength of interest from a broadband source, in order to offer a higher detection sensitivity [40,41]. Another implementation featured the detection of CO${}_{2}$ based on plasmonic emitters and detectors, which significantly modify the level of absorption in the window of interest [42]. Based on its electric field-to-current conversion capabilities, the metasurface layer can also be used directly as a detector or by layering it to another pyroelectric substrate. In addition, when used in reflection mode, metasurface-based gas sensors offer a direct visual alarm if the gas concentration levels exceed certain levels [43]. The proposed architecture consists of a combination between a liquid crystal layer and a metasurface. The presence of a volatile gas changes the orientation of the liquid crystal moelcules across a certain thickness and therefore modifies the optical activity of the structure. The chirality of the metasurfaces induces a certain optical reflection response, which can be holographically tuned to a readable alarm signal. In infant stages of development, metasurfaces were constructed using metallic shapes deposited on dielectric substrates, based on theoretical predictions such as Bruggeman [44] and Maxwell-Garnett [45,46] theories. However, due to significant energy loss in the metal host and incomplete phase coverage, such configurations have been used mostly for proof-of-principle demonstrations rather than standalone applications. All that changed with the introduction of hybrid [47,48] and all-dielectric [49] metasurfaces, which were able to circumvent most of the disadvantages of the all-metallic counterparts. By comparison to the classic (metalli(**c**) architecture, hybrid metasurfaces introduce a new degree of freedom in the meta-atom configuration, by establishing an imbalance between the permittivities $\epsilon_{j}$ and permeabilities $\mu_{j}$ of the *j* material elements of the cell. In practical setups, these hybrid structures are currently obtained by layer stacking on cascaded dielectric substrates, but new methods offer the perspective of printing the metasurface elements directly onto the same substrate. Another limitation of metasurfaces is that once configured, they cannot be tuned out of the desired resonance. This induces a high resolution requirement in the manufacturing process, which is especially hard to realize in a reproducible manner for working wavelengths approaching the visible threshold. To lessen the burden of high resolution, as well as offer on-demand tunability, hybrid metasurfaces also employ either deformation-addressable materials such as piezoelectrics or MEMS [50] for operation in the far infrared and terahertz regimes or liquid crystals and their composites for operation in the near- and mid-infrared ranges [51,52,53]. The use of such systems in combination with metasurfaces offer added versatility and control of the input field, resulting in an enhancement of the desired response. While all-dielectric metasurfaces extend the interaction capabilities of the cell and minimize loss by heat dissipation, the patterning techniques for the near-infrared regions of the spectrum remain challenging, mostly due to the inability to recreate the periodic pattern over multiple cell arrays with a high-enough resolution. Given the current state of the art, a good compromise between loss, resolution and phase coverage can be obtained in hybrid metasurfaces.

In this paper, we theoretically investigate the electromagnetic interaction response of a hybrid gold-silicon cell with a new asymmetric architecture, by comparison to an all-silicon counterpart. We stress that, while extensive studies were theoretically and experimentally performed on ring-based metasurfaces with different configurations and materials [54,55,56], the structure configuration was previously obtained by layering different geometries on top of each other, which led to significant signal loss and aberrations due to defects in the volume of the layers. The proposed in-plane deposition technique of elements with different geometries and different materials introduces a new degree of freedom to the structure and modifies the resonant frequency of the structure to frequencies that would have been otherwise obtained with much smaller geometric dimensions. This technique is technologically enabled by nano-imprint lithography (NIL) and nano-stencil lithography (NSL), which are able to design sub-micrometer structures of given shapes and materials in the same working plane [57]. The gold-silicon cell configuration exhibits frequency-selective behavior in the window of interest for cell element sizes of roughly λ/5, which challenges the rule of thumb that states that typically the cell element sizes have to be around λ/10 in order for the cell to be considered a metasurface [7]. We compare the spectral behavior of the metasurface cell with the properties of the individual gold and silicon elements and discuss the implications on possible applications. We also investigate the effect of stability of the tuning curve for resonance as well as dielectric investigations that permit the determination of the effective permittivity of the metasurface.

## 2. Architecture Design and Simulation Conditions

**Figure 1:**
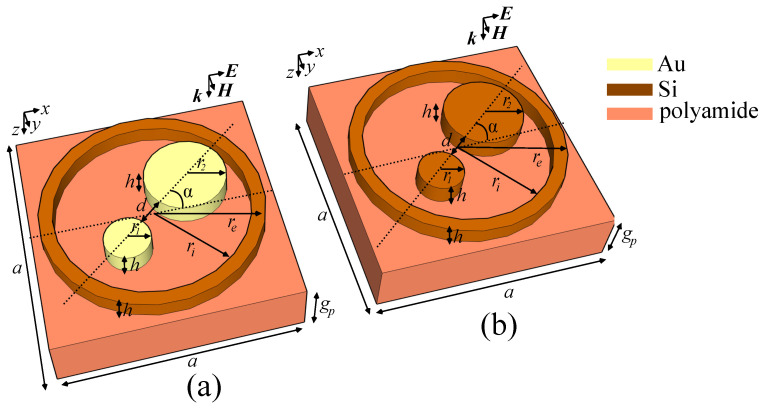
Layout of the proposed metasurface unit cell in (**a**) the silicon-gold and (**b**) the all-silicon configurations. The two cylinders with radii $r_{1}$ and $r_{2}$ are placed with the centers along an axis rotated with an angle α with respect to the Ox axis.

Our proposed metasurface highlights the use of the nonlocal approach in the simulation. Firstly, the designated material properties also include both spectral response and loss coefficient (where necessary), and we impose certain modifications on the boundary conditions: contrary to the more computing-efficient transition layer, which establishes an a priori averaging of the material properties across the thickness of the interface, we performed an exact simulation on the field impinging on a three-dimensional interface. Moreover, the Floquet periodicity condition that we imposed on the boundary was set to determine the fundamental propagation modes, filtering out the higher-order Bragg modes. The strong nonlocality of our metasurface is also emphasized in the field and phase maps taken at appropriate wavelengths in the plane pependicular to propagation. In addition, to illustrate the novel behavior of the cell and to highlight the feasibility under reduced manufacturing costs, in our simulations we only used readily-available materials such as polyamide, silicon and gold. The reference configuration model of the hybrid cell consists of a silicon ring with an external radius $r_{e}=2\;\mu$m, an internal radius $r_{i}=1.5\;\mu$m, a relative permittivity $\epsilon_{r,Si}=11.7$ and relative permeability $\mu_{r,Si}=0.9983$. Inside the ring, there are two cylindrical parts, having radii $r_{1}=0.4\;\mu$m and $r_{2}=0.6\;\mu$m centered on a diameter axis of the ring in such a way as to create a gap d=1.4μm between them. The ring and cylinders are made of either gold or silicon, depending on the desired study. For the gold-based configuration, the electrical conductivity value that was used is $\sigma_{Au}=45.6\;S/\mu$m. The diameter axis that contains the centers of the two cylinders is also inclined at an angle α with respect to the *Ox* axis, offering another degree of freedom. For the reference configuration, we chose $\alpha =0^{\circ }$. All the components are situated on a parallelepipedic polyamide substrate having a rectangular surface with dimension a=5μm thickness $g_{p}=0.2\;\mu$m and complex permittivity $\epsilon_{p}=2.88-0.09i$. Due to the fact that dispersion properties of the bulk material components induce little variation in the values of both ϵ and μ across the designated spectrum, the values of $\epsilon_{r}$ and $\mu_{r}$ are considered constant. This helps improve the calculation time, while maintaining the accuracy of the solution. All deposited components have the same height h=0.5μm. The depiction of the cell model is given in Figure 1. In order to establish the spectral response, we performed simulations of the reference cell composed of several material combinations, and we have varied several parameters in order to establish the cell sensitivity to tuning.

In terms of simulation conditions, all simulations were performed using a commercial Finite Element Time Domain (FETD)-based simulation medium—COMSOL Multiphysics, Radio Frequency (RF) interface. We initially assumed a normally incident, linearly polarized plane wave, with the electric field E‖Ox, the magnetic field H‖Oy and k‖Oz, having the input power in the low-value regime. This regime is selected in order to account for a linear response of the structure, without any third-order nonlinearity deviation. The interface was programmed to execute computations of the spatial component of the electromagnetic wave equation:

(1) $$\nabla \times \mu_{r}^{-1}\left(\nabla \times E\right)-k_{0}^{2}\left(\epsilon_{r}-\frac{j\sigma }{\omega \epsilon_{0}}\right)E=0$$

where $j^{2}=-1$ is the imaginary part of the complex number set, assuming a set of standard constitutive relations for each field. To simulate propagation and detection in the far-field, we assumed a rectangular box filled with air, with a propagation length $L\gg h+g_{p}$. The metasurface was placed at a coordinate y=L/2, with its director parallel to the long axis of the box. The input and output ports were taken at the upper and lower facets of the air box. In addition, in terms of boundary conditions, to eliminate parasitic scattering on the box walls, a perfect electric conductor (PEC) condition was used on the air box facets oriented parallel to *Ox*, while a perfect magnetic conductor (PMC) condition was used on the air box facets oriented parallel to *Oy*. To account for the periodicity of the cell, a Floquet periodicity condition (FP) was used on the cell facets oriented parallel to Ox and Oy, where the Floquet wave vector was calculated iteratively from the values obtained at the edges of the cell. The FETD solver was set to resolve the frequency domain behavior of the structure, in the stationary regime (no transient effects were considered). The floating point accuracy of the solver was set as 0.1%, as a standard consideration, and the meshing dimension of the entire geometry was set to approximately λ/50, with a λ/100 resolution in the interaction regions.

## 3. Results and Discussions

**Figure 2:**
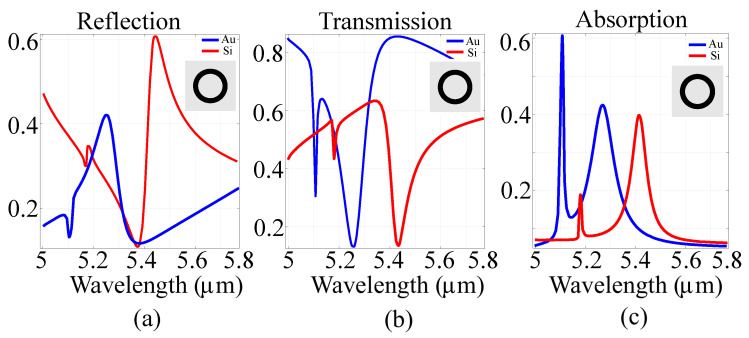
(**a**) Reflection, (**b**) transmission and (**c**) absorption of the ring architecture for a set of fixed parameters, using silicon and gold as materials. The inset presents the layout of the cell in the *xy* plane.

To establish the contribution of the composing elements on the spectral behavior of the ensemble, and to certify the modified spectral response depending on the architecture, we first performed simulations on the architecture containing just a silicon ring and compared to its metallic counterpart. We then proceeded to insert the metallic cylinders inside the silicon ring and compared the resulting architecture with a full-silicon counterpart. The spectral response of the designed architectures was investigated in the far-field, in terms of intensity transmission, reflection and absorption coefficients. As a first step, the architecture consisting of just the ring was considered, and the results are presented in Figure 2.

**Figure 3:**
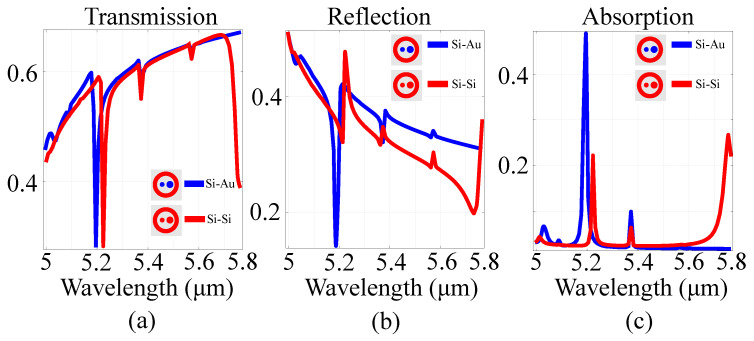
(**a**) transmission, (**b**) reflection and (**c**) absorption of the all-silicon and gold-silicon metasurface cells in the spectral regions of interest. The inset presents the layout of the cell in the *xy* plane. The red color in the inset indicates silicon, while the blue color indicates gold.

Based on the results, a frequency-selective behavior was observed for both materials, with resonances at 5.1μm and 5.28μm in the case of the gold ring. For the silicon ring, resonances shifted to 5.19μm and 5.42μm, with an associated change in absorption amplitudes. Moreover, in the metallic architecture, changing the material from gold to silver, copper or tungsten does not yield any considerable shift. This is due to the fact that the value of the electric conduction of the metallic parts does not change significantly. Moreover, since metallic metasurfaces exhibit significant heat dissipation losses and weak phase coverage, they are mostly used as proof-of-principle demonstration. In devices, either semiconductor or hybrid semiconductor-metal architectures are preferred. To wit, we have performed spectral investigations on the full structure presented in the previous section, in two situations: one where all parts are constructed out of silicon and another where the ring is constructed out of silicon and the inner cylinders are constructed out of gold. The spectral behavior is presented in Figure 3.

From the data, it can be observed that the change in transmission and reflection is significant in the two configurations, with two pronounced resonance peaks in the 5–5.6 μm window. This behavior is dictated by the ring geometry rather than the materials used. However, in the silicon ring configuration, the response features a new resonance peak at approximately 5.8μm, along with a relative shift in the resonance peaks. For both reference cell configurations, the absorption profiles exhibit multiple symmetric, Lorentzian-shaped resonances [58]. The multiple sharp peaks in the absorption spectrum for the hybrid silicon-gold architecture can also be attributed to the hybrid meta-molecule, which combines the continuous transition band of gold with the discrete interband of silicon [59].

**Figure 4:**
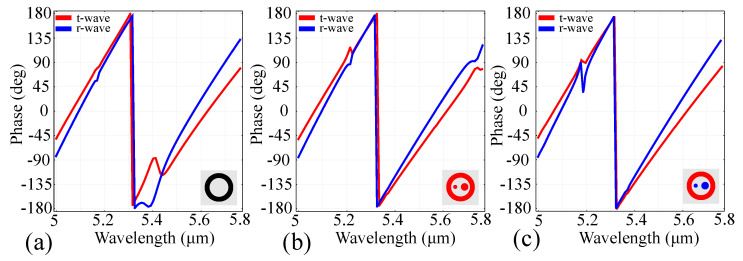
Phase coverage across the window of interest in the reference configuration for: (**a**) the silicon ring; (**b**) the all-silicon configuration; (**c**) the gold-silicon configuration. The configurations are indicated in the insets, with silicon being represented in red and gold represented in blue.

We have also conducted simulations that highlight the phase coverage of the metasurface, and the results are presented in Figure 4.

The simulation results indicate that the ring architecture offers an almost full 2π coverage, with a smooth transition from 5μm to 5.31μm, followed by a sharp transition from almost $+180^{\circ }$ to $-180^{\circ }$, and a similar smooth transition afterwards. The ring-cylinder configurations modify this behavior only slightly, by providing a relatively-small shift of the sharp transition towards a higher wavelength, and linearizing the smooth transition behavior. In the all-silicon configuration, the linear transition is deformed close to 5.7μm for all waves, while in the gold-silicon configuration the linear transition is deformed at around 5.2μm for both reflected and transmitted waves.

**Figure 5:**
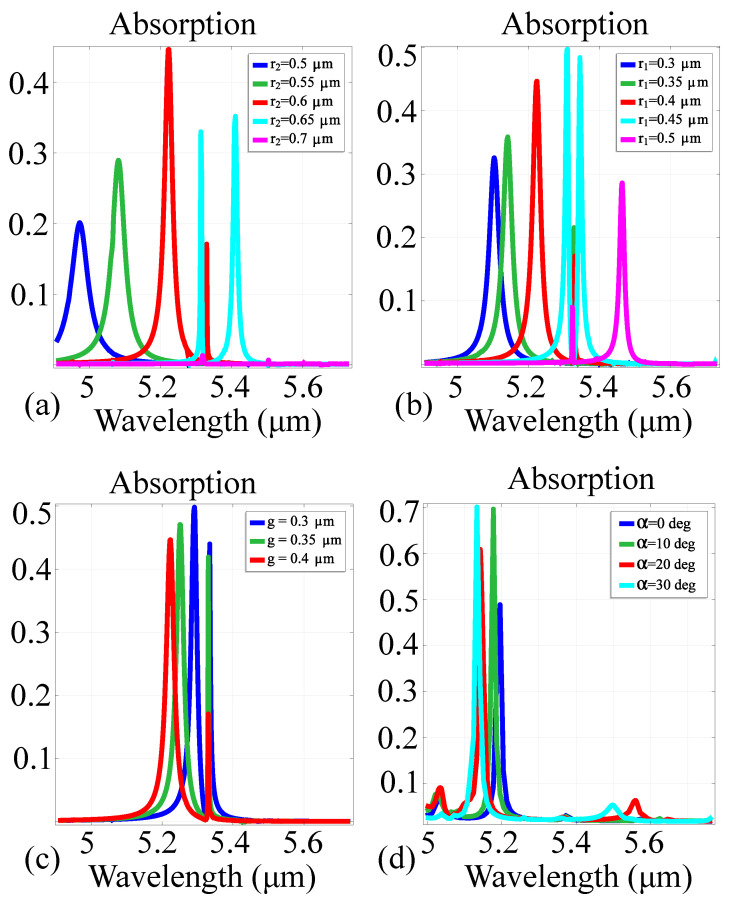
Absorption tuning curves of the hybrid silicon-gold cell as a function of various degrees of freedom: (**a**) tuning curves for a variation of the large cylinder radius $r_{2}$; (**b**) tuning curves for a variation of the small cylinder radius $r_{1}$; (**c**) tuning curves for a variation of the substrate thickness $g_{p}$; (**d**) tuning curves for a variation of the cylinder inclination axis angle α. For the variation of one parameter, all other ones have their values corresponding to the reference configuration.

To determine the tuning properties of the designed metasurface cell, we also performed resonance sensitivity simulations as a function of various geometric parameters in the hybrid silicon-gold cell architecture. The degrees of freedom taken into consideration were the radii $r_{1}$ and $r_{2}$ of each cylinder, the substrate thickness $g_{p}$ and the angle α between the symmetry axis of the two cylinders in the *xy* plane and the *Ox* axis. We reiterate that in the reference configuration, the values of the cell elements are $r_{1}=0.4\;\mu$m, $r_{2}=0.6\;\mu$m, $g_{p}=0.2\;\mu$m and $\alpha =0^{\circ }$. For any variation in cell element values, the rest of the parameters have their values fixed. The results are presented in Figure 5.

The obtained results highlight the following metasurface properties: firstly, as a general trend, the metasurface exhibits significant resonance over the majority of unit cell configurations, with values ranging between 0.2 and 0.5 of the incident field. For variable $r_{2}$ values, the metasurface exhibits either one or two resonance peaks. For $r_{2}=0.5\;\mu$m and 0.7μm, the response exhibits a single resonance peak, at 5μm and 5.15μm respectively. For $r_{2}=0.6\;\mu$m and 0.65μm, the response exhibits two resonance peaks, at 5.2μm and 5.32μm for $r_{2}=0.6\;\mu$m and at 5.35μm and 5.4μm for $r_{2}=0.65\;\mu$m. For $r_{2}=0.7\;\mu$m, there is relatively no resonant response in the window of interest. When modifying $r_{1}$, the following behavior is observed: For $r_{1}=0.3\;\mu$m and 0.4μm, there is a single resonance peak at 5.16μm and 5.22μm, respectively. For $r_{1}=0.35,0.45$ and 0.5μm, the response exhibits two resonance peaks. These peaks are located at 5.19μm and 5.35μm for $r_{1}=0.35\;\mu$m. For $r_{1}=0.45\;\mu$m, the resonance peaks are closer together, located at 5.3μm and 5.35μm. In addition, for $r_{1}=0.5\;\mu$m, the resonance peaks are shifted towards higher wavelengths and further apart, at 5.35μm and 5.47μm. When varying the substrate thickness, the two resonance peaks observed at 5.22μm and 5.35μm for $g_{p}=0.4\;\mu$m are shifted closer together as $g_{p}$ decreases. Moreover, the absorption peak at 5.35μm increases its value from 0.17 in the reference configuration to 0.45 in $g_{p}=0.3\;\mu$m. When varying α between fixed values, the resonance peaks are shifted towards smaller wavelengths. The peak obtained at 5.22μm is shifted towards 5.18μm and its value is increased from 0.5 to approximately 0.7. Simultaneously, the resonance peak at 5.35μm is very slightly shifted towards smaller wavelengths, with a decrease from 0.17 to 0.09 of the incident wave. Moreover, in some configurations, the absorption response exhibits certain asymmetry, as is the case in the $g_{p}=0.4\;\mu$m configuration. The asymmetric shape of the peak at 5.37μm indicates the presence of Fano resonances [60].

**Figure 6:**
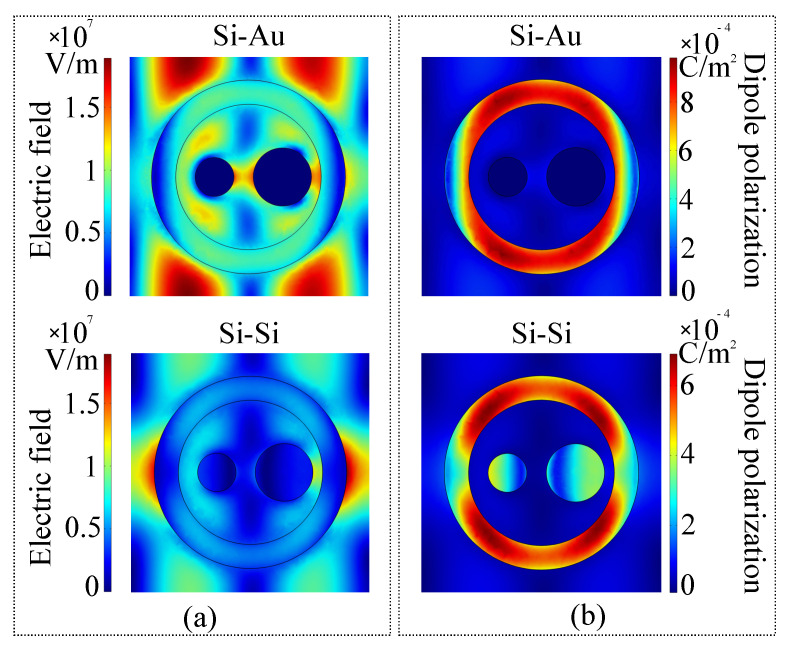
(**a**) Electric field norm and (**b**) induced dipole polarization in the metasurface plane taken at the dominant resonance frequency of the gold−silicon and all−silicon configurations.

With the desired resonance tuning effect established, we also performed standard electromagnetic and optical wavefront characterization for both the hybrid and all-silicon metasurface in the reference configuration. In terms of simulation conditions, the plane of interest was taken at the interface between the metasurface and exterior, and the simulations were conducted at the higher-valued resonance frequency of each architecture. The motivation for this configuration is the desire to observe the existence of possible electric multipoles, that are known to contribute to a modified scattering efficiency [61]. The electric field parameters considered are the electric field and induced dipole polarization, and the results are presented in Figure 6.

**Figure 7:**
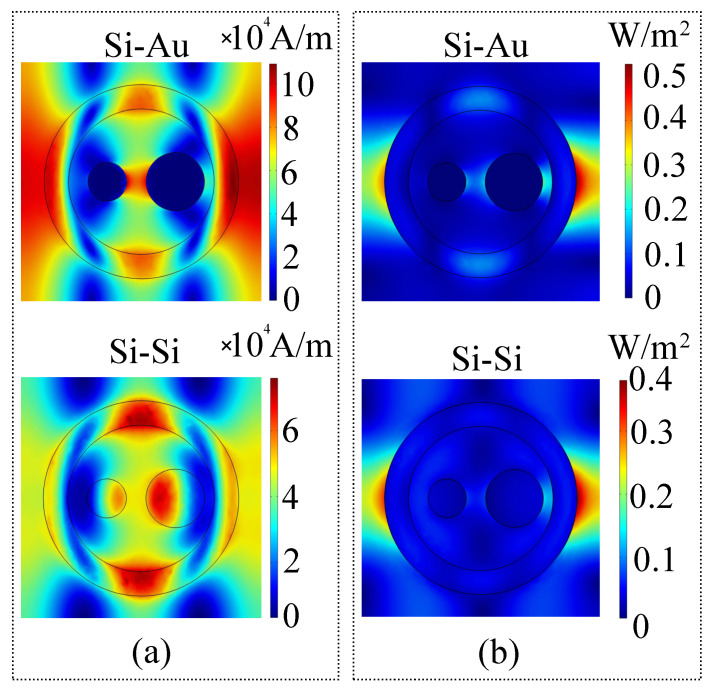
(**a**) Magnetic field and (**b**) average power flow of the wave induced by the metasurface elements in the gold-silicon and all-silicon configurations.

Regarding the silicon-gold metasurface, the symmetry of the electric field norm outside the ring indicates the presence of a quadrupolar moment, induced by the symmetry of the ring. Inside the ring, a relatively-high amplitude electric field is observed, confirming the existence of nanoplasmonic resonance. As expected, there is no electric field inside the gold disks, confirming once again the validity of the simulation. The dipole polarization is concentrated around the ring, which further reinforces the idea that this structure is responsible for multipolar resonances. In the case of the all-silicon configuration, the lateral lobes of the multipolar resonance are amplified, while the lobes of the quadrupolar moment are attenuated. This behavior is consistent with previously reported octupolar moments that appear in all-dielectric nanoplasmonic resonators [61]. Similarly, the magnetic field, average power flow and phase variation in the metasurface plane were simulated, and the results are presented in Figure 7.

As the simulation data indicates, the multipolar resonance of the magnetic field distribution reveals the same quadrupolar symmetry for the silicon-gold configuration, whereas for the all-silicon configuration, the magnitude of the field is maximal in the ring and disk structures. The average power flow distribution serves as another simulation validation, as it follows the direction of the electric field. This is to be expected seeing as how the electric field is naturally of a much larger magnitude than the magnetic field. Moreover, the average power flow and phase variation are not changed significantly when interchanging configurations, which indicates that the switch between a hybrid and an all-silicon version will not change the direction and polarization of the wave. It can be observed that due to the degree of freedom induced by angle α, the metasurface is also a nonlocal 2π phase-changing element.

## 4. Conclusions

In this paper, we have presented theoretical comparative investigations between a gold-silicon and all-silicon metasurface in the mid-infrared regime, obtaining a tunable frequency-selective behavior. The surface exhibits qualitative electromagnetic and optical properties, with relatively-high absorption in the desired spectral window and with a 2π control over the phase for the reflected and transmitted waves. The response of the proposed metasurface architecture allows its use in mid-infrared applications such as infrared wave controllers in transmission and/or reflection, signal detectors when used in combination with a pyroelectric substrate, gas sensors when used as tunable bandpass filters and frequency-selective surfaces for atmospheric and LIDAR applications.

## Data Availability

Not applicable.
